# Protective effects of *Persea americana* fruit and seed extracts against chemically induced liver cancer in rats by enhancing their antioxidant, anti-inflammatory, and apoptotic activities

**DOI:** 10.1007/s11356-022-18902-y

**Published:** 2022-02-04

**Authors:** Osama M. Ahmed, Hanaa I. Fahim, Eman E. Mohamed, Adel Abdel-Moneim

**Affiliations:** 1 Physiology Division, Zoology Department, Faculty of Science, Beni-Suef University, Beni Suef, Egypt; 2 Molecular Physiology Division, Faculty of Science, Beni-Suef University, Salah Salem St, Beni Suef, 62511 Egypt

**Keywords:** *Persea amricana*, Diethylnitrosamine, Acetylaminofluorene, Hepatocellular carcinoma, Inflammation, Apoptosis

## Abstract

This study aims to explore the chemopreventive mechanisms of hydroethanolic extracts from avocado (*Persea Americana*) in diethylnitrosamine (DEN)/2-acetylaminofluorene (2AAF)-induced hepatocarcinogenesis. Chemical induction of hepatocarcinogenesis was induced by intraperitoneal injection of DEN at 150 mg/kg body weight (b.w.) twice a week for a fortnight, followed by oral administration of 2AAF at 20 mg/kg b.w. four times a week for 3 weeks. Rats administered DEN/2AAF were orally treated with hydroethanolic extracts of avocado fruits and seeds at a dose of 50 mg/kg b.w. every other day for 20 weeks. Moreover, rats administered DEN/2AAF and treated with avocado extracts revealed a marked decrease in liver enzyme activities, total bilirubin levels, and elevated liver tumor markers, but revealed an increase in total protein and albumin levels. The hepatocytes with hyperchromatic and bile duct cystadenoma observed in the liver of rats administered DEN/2AAF were reduced due to treatment with avocado extracts. Furthermore, the treatments prevented the elevation of lipid peroxidation levels and ameliorated the lowered glutathione peroxidase, glutathione-S-transferase, superoxide dismutase activities, and glutathione content in the liver tissues. Also, antigen Ki-67, cyclooxygenase-2, and nuclear factor kappa-B expression levels were decreased, but of the suppressor proteins p53 and BAX levels were increased in the liver of rats administered DEN/2AAF and treated with avocado extracts. In conclusion, the current results demonstrated that avocado extracts could abate hepatocarcinogenesis in rats administered DEN/2AAF through activation of antioxidant, anti-inflammatory, and apoptotic properties.

## Introduction

Hepatocellular carcinoma (HCC) is the fifth most common cancer in the world and the second leading cause of mortality from cancer (World Health Organization. Global health observatory 2018) and it has been estimated to be responsible for nearly 9.1% of the total deaths (746,000 deaths) (Ferlay et al. 2015). Hepatocarcinogenic and toxic effects of diethylnitrosamine (DEN) are validated animal model (Arboatti et al. 2018). DEN is found in pharmacological compounds, cosmetics, tobacco smoke, and cured and cooked meals (Dar et al. 2019). DEN was activated by cytochrome P450 enzymes, resulting in reactive electrophile species that produce oxidative stress, cytotoxicity, and carcinogenicity (Rajkapoor et al. 2005). Accordingly, DEN was shown to cause the production of free radicals, oxidative stress, and cell damage, and altering DNA structure (Bansal et al. 2000; Al-Rejaie et al. 2009). Interestingly, DEN was conducted to induce HCC, while 2-acetylaminofluorene (2AAF) has been employed as a carcinogen promoter (Lto et al. 2003).

The antioxidants reduce the levels of oxidative stress via reactive oxygen species (ROS)-scavenging mechanisms (Simunkova et al. 2019). Lipids, nucleic acids, and proteins may be damaged by ROS, thereby altering their functions. Oxidative stress occurs when the equilibrium between ROS development and antioxidant protection is disrupted (Jelic et al. 2020). Unregulated and continuous liver imbalances among ROS production and ROS removal by protective mechanisms (antioxidants) lead to chronic disease and damage to vital biomolecules and cells (Reyes-Gordillo et al. 2017). Phytonutrients are herbal nutrients or phytochemicals with possible health benefits to preserve the well-being and normal functions of the body and to improve life expectancy (Memariani et al. 2020).

Avocado is a Central American tree; its fruits are widely used as a nutrition source and treatment of diseases. They have many biological benefits, like cholesterol-lowering, analgesic, and anti-inflammatory effects (Nicolella et al. 2017). Avocado fruit has nutrient contents such as vitamin B9, vitamin B6, vitamin C, vitamin K, dietary fiber, potassium, and copper (Dreher and Davenport 2013). It is commonly cultivated and eaten worldwide and has several health benefits (Mahmassani et al. 2018). In addition, avocado fruit contains several phytochemicals that have potential anticancer activity (Lu et al. 2005) such as avocation B which reduced human primary acute myelogenous leukemia without affecting normal peripheral blood stem cells (Lee et al. 2015). Avocado has been used in herbal medicine as an anticancer and was studied as a hepatoprotective agent (Mahmoed and Rezq, 2013). The anti-cancer effects of avocado seeds (lipid-rich extract) were reported in vitro against various types of cancer cell lines including Caco-2 (Lara-Márquez et al., 2020), HCT116, and HePG2 (Alkhalaf et al., 2019). Moreover, Abozaid et al. (2018) stated in his preliminary study that avocado oil could be considered a promising therapeutic adjuvant against DEN-induced hepatocarcinogenesis. The protective anti-carcinogenic mechanisms of avocado hydroethanolic extracts in the DEN/2AAF-induced liver cancer model are not fully explored. Thus, our study aims to assess the anti-carcinogenic effect and explore the mechanisms of hydroethanolic extracts from avocado fruits and seeds in DEN/2AAF-induced hepatocarcinogenesis in Wistar rats.

## Materials and methods

### Experimental animals

Male adult Wistar rats, weighing 100–120 g, were included. The rats were purchased from the Egyptian Organization for Biological Products and Vaccines (VACSERA) Animal House Facility in Helwan, Cairo, Egypt. They were then kept under observation for 14 days before the beginning of the study to eliminate any infections. The rats were kept in polypropylene cages, with well-aerated stainless steel covers at normal temperature (20–25 °C) and regular daylight cycle (10–12 h/day) and a well-balanced standard diet ad libitum. All animal experiments were conducted in compliance with the general guidelines of animal care and the recommendations of the Experimental Animal Ethics Committee of Faculty of Science, Beni-Suef University, Egypt (BSU/FS/2015/6).

### Chemicals and preparation of extracts

DEN (#N0756) and 2AAF (#A7015) were obtained from Sigma Chemicals Co. (St. Louis, MO, USA) and stored at 2–4 °C. Other chemicals were classified as of analytical grade. In November, avocado pear (*Persea americana*) fruits were obtained from a local market in Beni Suef, Egypt. They were authenticated by Prof. Dr. Mohamed Ahmed Fadl, Professor of Taxonomy, Botany Department, Faculty of Science, Beni-Suef University, Egypt.

Seeds have been isolated from fruits (pulps and peels), according to Ilochi and Chuemere (2019). In a well-aerated shade field, fruits and seeds were cut into pieces, dried for 15 days before being coarsely powdered, and then extracted with 1 l of 70% ethanol (1:2 w/v) for 72 h at room temperature (25 °C). The hydroethanolic extract from fruits and seeds was filtered with Whatman filter paper and concentrated by a rotary evaporator. The yield was determined to be 1%, and the extracts were stored in glass bottles at − 18 °C, until used.

### Gas chromatography–mass spectrometry analysis

Gas chromatography-mass spectrometry (GC–MS) is an analytical method that combines gas-chromatography and mass spectrometry features to identify different substances within a test sample. Additionally, it can identify trace elements in materials that were previously thought to have disintegrated beyond identification. It allows the analysis and detection of tiny amounts of a substance (Sparkman et al. 2011). A 7890A/5975C Inert MS-GC system with triple Axis Detector, Agilent Technologies (Germany), was used to analyze chemical constituents of avocado fruit and seed hydroethanolic extracts at the Central Laboratory of the Faculty of Postgraduate Studies for Advanced Sciences, Beni-Suef University, Egypt. The splitless mode was used to inject the hydroethanolic extract of fruits and seeds at a volume of 1 µl. A temperature of 250 °C was kept in the injection port. The oven temperature program begins at 120 °C and gradually rises to 220 °C at a rate of 5 °C/min, followed by 8 °C/min to 280 °C for 5 min. Helium gas was utilized as a carrier gas at a flow rate of 1.0 ml/ min, and the total run time was 32.5 min. The components were identified by comparing their mass spectra to the derivative spectra in the Library Search Report (C:\Database\NIST11.L; C:\Database\demo.l).

### Experimental design

Four groups of ten adult male Wistar rats were allocated. The normal control group was Group I, while the other three groups were administered DEN intraperitoneally twice a week for a fortnight at a dose of 150 mg/kg body weight (b.w.), accompanied by oral gavage treatment with 2-AAF (20 mg/kg b.w.) four times a week for 3 weeks (De Lujan Alvarez et al. 2002). Group II received DEN/2AAF and served as a positive control group, and groups III and IV received DEN/2AAF and were simultaneously treated orally with avocado fruit and seed, respectively. Groups III and IV were each treated with avocado fruit and seed hydroethanolic extracts at a dose level of 50 mg/kg b.w. (Monika and Geetha 2016; Abdel-Moneim et al. 2017) every other day for 20 weeks (total duration of the study). Both groups I and II received the same volume of the vehicle (5 ml 1% CMC) in which the avocado fruit and seed were dissolved.

Diethyl ether was used to anesthetize the animals before they were sacrificed at the end of the experiment. Blood samples were obtained from the jugular vein and allowed to coagulate, then centrifuged at 3000 rpm for 15 min; sera were collected in sterile tubes and kept at − 20 °C. After homogenizing 1 g of frozen liver tissue, 1% homogenate was produced in 10-ml phosphate buffer saline (pH 7.4). Liver homogenates were centrifuged for 15 min at 3000 rpm. The supernatant is isolated and held at − 20 °C until used to investigate oxidative stress and antioxidant biomarkers. A total of 3-mm^3^ liver pieces were stored at − 70 °C in sterilized tubes until used for RNA isolation and RT-PCR assay.

### Biochemical investigations

The activities of serum alkaline phosphatase (ALP) (#11,592), alanine transaminase (ALT) (#11,533), aspartate aminotransferase (AST) (#11,531), and gamma-glutamyl-transpeptidase (GGT) (#11,520) were estimated using reagent kits obtained from Biosystem S.A. (Spain). Serum total bilirubin (#10,740), total protein (#10,570), and albumin (#156,004) levels were measured using Human Diagnostics (Germany) reagent kits. Serum carbohydrate antigen 19–9 (CA19-9) (#MBS729408), carcinoembryonic antigen (CEA) (#MBS700529), and alpha fetoprotein (AFP) (#MBS034337) were assayed by Sandwich ELISA using kits from R&D Systems (USA) according to the manufacturer’s instructions.

The levels of lipid peroxidation (LPO) in liver homogenate were determined by measuring malondialdehyde (MDA) production using the technique of Yagi (1987). To summarize, the protein was precipitated by adding 0.15 ml of 76% trichloroacetic acid (#T6399, Sigma Chemicals Co., USA) to 1 ml of liver homogenate. Then, 0.35 ml of thiobarbituric acid was added, as a color-developing agent, to the separated supernatant. After incubation in a water bath at 80 °C for 30 min, the formed faint pink color was detected at 532 nm. The standard used was MDA (1,1,3,3-tetramethoxypropane). Following the procedure of Beutler et al. (1963), reduced glutathione (GSH) level in liver was evaluated by adding 0.5-ml 5,5'-Dithiobis (2-nitrobenzoic acid) (#D-8130, Sigma Chemicals Co., USA), Ellman’s reagent (as a color-developing agent), and phosphate buffer solution (pH 7) to the homogenate supernatant after protein precipitation. At 412 nm, the produced yellow color in the samples and GSH standard was compared to the blank. In addition, the activity of liver glutathione-S-transferase (GST) was measured in the presence of GSH and 1-chloro-2,4-dinitrobenzene (CDNB) dissolved in ethanol using the Mannervik and Guthenberg (1981) technique. The molar extinction coefficient of 9.6 mM-1 cm-1 was used in the calculations. In particular, 250 µl CDNB (4 mM) was added to a Wasserman tube containing 250 µl sample, 250 µl GSH solution (4 mM), and 250 µl phosphate buffer (pH 7.3). The developed color was assessed after 10 min of incubation at 25 °C.

The activity of liver glutathione peroxidase (GPx) was assessed using the Matkovics et al. (1998) method, which involves detecting the GSH that was converted to oxidized glutathione (GSSG) by the enzyme and subtracting it from the total. In a Wasserman tube containing 350-µl Tris buffer (pH 7.6), 50-µl GSH solution (#A2084, Applichem, Germany) (2 mM), and 50-µl H2O2 (3.38 mM), 50-µl homogenate supernatant was added. Then, after 10 min of incubation, the residual GSH content was determined at 430 nm using the previously reported technique for GSH measurement. The standard test was performed by adding 50-µl distilled water instead of 50-µl sample and the blank test was performed by adding 100-µl distilled water instead of 50-µl sample and 50-µl GSH solution. Following the detection of residual GSH in the sample, the GSH converted to oxidized form (GSSG) and enzyme activity was estimated. Furthermore, the activity of superoxide dismutase (SOD) in the liver was measured using the technique of Marklund and Marklund (1974). The process is based on SOD inhibiting the auto-oxidation of pyrogallol. The process is dependent on the presence of superoxide ions. One unit of enzyme is defined as the quantity of enzyme that inhibits extinction changes by 50% in 1 min when compared to the control. Briefly, 50 µl of pyrogallol (10 mM) was added to 1 ml of the homogenate supernatant in the presence of Tris buffer (pH 8). After adding pyrogallol, the initial absorbance was measured, as well as 10 min later. The enzyme activity and the inhibition of the formed yellow color at 430 nm were assessed. The biochemical investigation was measured by spectrophotometer, Humalyzer 3000, Human Diagnostics (Germany).

### RNA isolation and RT-PCR analysis in serum

The total RNA was extracted from the liver using a Thermo Scientific GeneJET RNA purification kit (#K0731) obtained from Thermo Fisher Scientific Inc. (Rochester, New York, USA) according to the method of Chomzynski and Sacchi (1987). A UV spectrophotometer was employed to determine the amount of extracted RNA. The purity of the extracted RNA was verified and must be ranged between 1.8 and 2.0. After that, 0.5 g of total RNA was added to produce cDNA. The produced cDNA was amplified using Thermo Scientific Verso 1-Step RT-PCR ReddyMix (#F-580L) obtained from Thermo Fisher Scentific Inc. (Rochester, New York, USA) and specific primers provided from Biosesrch Technologies, South McDowell Blud, Petaluma, CA, USA. Ten µl of PCR products was examined on a 1.5% agarose gel (#Bio-41026) (Bioline Reagents Ltd, UK) stained with ethidium bromide (#A1151) (Applichem, Germany) in 1X Tris Borate EDTA buffer (TBE) pH 8.3–8.5. The pair sequences of the following primers were used in this study: COX-2 was 5́-AGACAGATCATAAGCGAGGAC-3´ (forward) and 5́-CACTTGCATTGATGGTGGCTGT-3´(reverse) (Reuter et al. 1996; Ahmed et al. 2019) and NF-κB was 5'-AATTGCCCCGGCAT-3' (forward) and 5'ATGCGCCAATGCCCT-3′ (reverse) (Habibi et al. 2016), while the primer sequences for p53 were 5′-GCTGCCCTCCCTTC TCCTAG-3' (forward) and 5′-CCCCGACTTTGGAGTAGTCTGA-3′ (reverse) (Abdel-Moneim et al. 2021). The primer sequences of β-actin were 5′-TCACTATCGGCAATGTGCGG-3′ (forward) and 5′-GCTCAGGAGGAGCAATGATG-3′ (reverse) (Abdel-Moneim et al. 2021). The qPCR was performed, and the amplified data were analyzed using Livak and Schmittgen’s (2001) method. The electrophoretic image was visualized using a gel documentation system, and the results and the density of bands of the detected gene were calculated relative to β-actin.

### RT-PCR analysis for miR-122

MiRNeasy Mini kit (Qiagen, Düsseldorf, Germany) was used to extract total RNA from serum. Nanodrop and agarose gel electrophoresis (#1,613,004) (Bio-Rad, USA) was performed to assess the amount and quality of RNA. The miScript II RT cDNA synthesis kit (#4,366,596) (Qiagen, Düsseldorf, Germany) synthesized cDNA from total RNA (1 µg) following the manufacturer’s instructions. Additionally, each cDNA has been employed as a template for quantitative real-time RT-PCR and a primer specific for miR-122, F: 5′-TTGAATTCTAACACCTTCGTGGCTACAGAG-′3 and R: 5′-TTAGATCTCATTTATCGAGGGAAGGATTG-′3, for U6, F: 5′CTCGCTTCGGCAGCACA-′3 and R: 5′-AACGCTTCACGAATTTGCGT-′3. All reactions were performed on step one plus (Applied Biosystem). The quantity of PCR was normalized to that for housekeeping gene U6. The 2 − ΔΔCt method was used for the relative quantification. The data are calculated as fold change differences compared to the relevant controls.

### Histopathological and immunohistochemical investigations

Liver specimens from each animal were excised and decapitated by local dislocation and dissection of the head. After fixation in 10% neutral formalin buffered for 24 h, pieces were handled with paraffin embedding procedure, dehydrated with ethyl alcohol, xylene clearing, and paraffin immersed. Liver pieces (5 mm^3^) were embedded in paraffin wax at 60 °C. Sections of 4 micron thickness by slide microtome were stained with hematoxylin and eosin following Banchroft et al. (1996). In a microwave oven, tissue sections were incubated in a citrate buffer (pH 6.0) for antigen recovery. Peroxidase activity in phosphate buffer saline (pH 7.4) was quenched with 3% H_2_O_2_ for 10 min. After 10–15 min of incubation with 10% normal goat serum, non-specific binding sites were blocked. Sections were subsequently incubated with the primary antibody of Bax (#A0207) or Ki-67 (#A11390) (ABclonal Inc., China) overnight at 4 °C (diluted 1:200), then added two to three drops of the secondary antibody, horseradish peroxide-Polymer anti-Goat IgG, and were incubated at room temperature for 20 min. Diaminobenzidine was employed in a color reaction to visualize peroxidase activity, and the sections were counterstained with hematoxylin. The intensity of immunohistochemistry staining for each marker was digitally recorded using image slave software (ImageJ) (Abramoff et al. 2004).

### Statistical methods

Results were presented as mean ± standard error. The one-way analysis of variance was used to determine statistical differences between groups (SPSS version 20 software, Chicago, IL, USA), followed by Duncan’s test to compare different groups with significance set at *P* < 0.05.

## Results

**Fig. 1:**
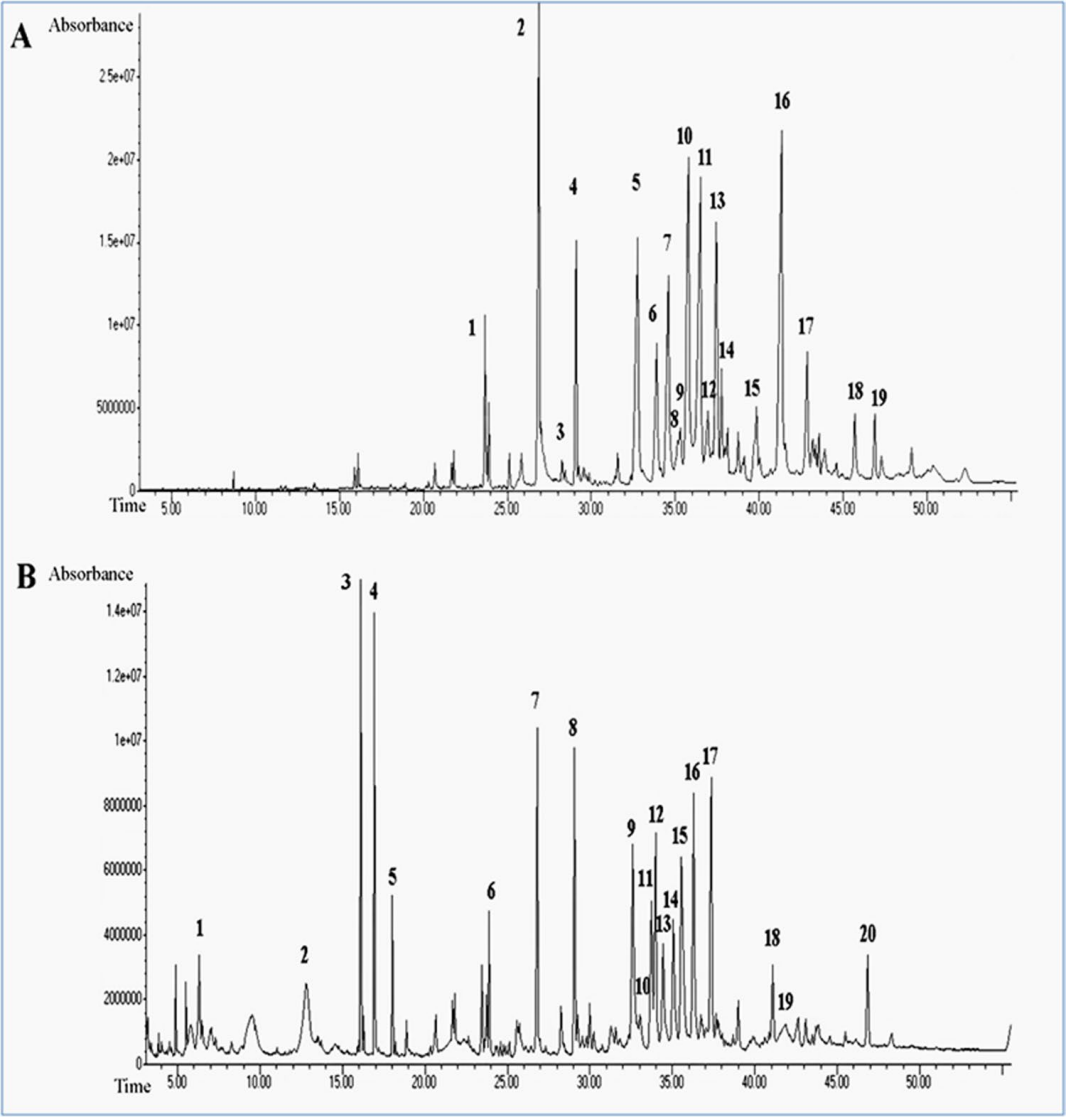
GC–MS analysis of **A** avocado fruit and **B** avocado seed hydroethanolic extracts

GC–MS analysis of hydroethanolic extracts from avocado fruit and seed showed phytocomponents. The described phytocomponents are summarized in Tables 1 and 2 and shown in Fig. 1 (a and b) with their molecular weight (MW), molecular formula (M/F), retention time (RT), and relative abundance represented as peak area % and activity.

**Table 1:** The chemical profile of the avocado fruit hydroethanolic extracts as identified by GC–MS analysis

No	RT	Name of the compound	Molecular formula	MW	Peak area %
1	23.677	7-Hexadecyne	C_16_H_30_	222.41	1.925%
2	26.879	No matches found			6.994%
3	27.003	3,7,11,Trimethyl-8,10- dodecedienyl acetate	C_17_H_30_O_2_	266.4	1.069%
4	29.088	No matches found			2.799%
5	32.772	No matches found			6.625%
6	33.888	No matches found			3.333%
7	34.594	No matches found			5.377%
8	35.145	2-Methyl-3-(3-methyl-but-2- enyl)-2-(4-methyl-pent-3-enyl)-oxetane	C_15_H_26_O	222.37	1.116%
9	35.302	No matches found			1.189%
10	35.785	9-Octadecyne	C_18_H_34_	250.5	8.988%
11	36.517	No matches found			9.368%
12	36.956	Cyclopropanemethanol	C_11_H_20_O	168.2	2.127%
13	37.45	No matches found			5.794%
14	37.767	No matches found			1.811%
15	39.851	3-Undecanol	C_13_H_28_O	200.3	1.448%
16	41.334	Ethanol, 2-(9,12-octadecadienyloxy)-	C_20_H_38_O_2_	310.5	9.081%
17	42.86	No matches found			2.854%
18	45.693	6-Tetradecyne	C_14_H_24_	194.3	1.302%
19	46.886	Bicyclo[10.1.0]tridec-1-ene	C_13_H_22_	178.3	1.059%

**Table 2:** The chemical profile of the avocado seed hydroethanolic extract as identified by GC–MS analysis

No	RT	Name of the compound	Molecular formula	MW	Peak area %
1	6.31	5-Hydroxymethylfurfural (5-HMF)	C_6_H_6_O_3_	126.11	1.729%
2	12.81	No matches found			2.545%
3	16.12	2-n-Heptylfuran	C_11_H_18_O	166.2	3.82%
4	16.936	No matches found			3.43%
5	18.03	No matches found			1.295%
6	23.891	No matches found			1.317%
7	26.808	3,7,11,Trimethyl-8,10- dodecedienyl acetate	C_15_H_32_O	225.4	3.641%
8	29.061	No matches found			3.301%
9	32.615	No matches found			4.56%
10	33.041	9-Octadecenamide	C_18_H_35_NO	281.4	1.135%
11	33.731	No matches found			2.977%
12	33.986	9-Octadecyne	C_18_H_34_	250.5	3.672%
13	34.435	No matches found			2.75%
14	35.063	6,11-Undecadiene	C_16_H_28_O_2_	252.3	3.22%
15	35.55	No matches found			5.246%
16	36.301	No matches found			4.917
17	37.359	Cyclopropanecarboxylic acid,undec-10-enyl ester	C_15_H_26_O_2_	238.37	4.661%
18	41.086	13-Tetradece-11-yn-1-ol	C_14_H_24_O	208.3	1.476%
19	41.836	gamma-Sitosterol	C_29_H_50_O	414.7	1.677%
20	46.836	Bicyclo[10.1.0]tridec-1-ene	C_13_H_22_	178.1	1.383%

### Effect on liver function

Wistar rats administered DEN/2AAF for 20 weeks observed significant (*P* < 0.05) increases in serum AST, ALT, GGT, and ALP activities and total bilirubin levels, while marked (*P* < 0.05) decreases in total protein and albumin levels were recorded compared to the normal group. Rats administered DEN/2AAF and treated with hydroethanolic extracts of avocado fruits and seeds showed a noticeable decrease in the elevated activities of serum ALP, GGT, and ALT, and levels of total bilirubin compared to rats administered DEN/2AAF only. The treatment with avocado seed extract induced a marked increase in total serum protein concentrations, while the administration of avocado fruit extract showed a noticeable increase in serum albumin levels (Table 3).

**Table 3:** Effect of avocado fruit and seed hydroethanolic extracts on serum parameters related to liver function in rats administered DEN/2AAF

Group	ALT (U/L)	AST (U/L)	ALP (U/L)	GGT (mU/dl)	Total bilirubin (mg/dl)	Total protein (g/dl)	Albumin (g/dl)
Normal group	44.5 ± 2.07^a^	132.8 ± 5.20^a^	157.6 ± 17.01^a^	18.00 ± 2.73^a^	0.56 ± 0.02^a^	8.6 ± 0.32^b^	4.21 ± 0.10^b^
DEN/2AAF-CG	66.5 ± 4.31^b^	156.5 ± 4.86^b^	310.8 ± 17.48b	36.33 ± 1.49 ^b^	0.84 ± 0.08^b^	6.7 ± 0.15^a^	3.73 ± 0.04^a^
DEN/2AAF + AFE	51.3 ± 5.30^a^	139.5 ± 9.17^ab^	208.5 ± 16.05^a^	21.33 ± 0.76 ^a^	0.64 ± 0.04^a^	7.8 ± 0.47^ab^	4.16 ± 0.15^b^
DEN/2AAF + ASE	52.5 ± 3.23^a^	152.0 ± 2.58^b^	206.0 ± 22.37^a^	23.16 ± 1.49^a^	0.60 ± 0.01^a^	8.0 ± 0.44^b^	4.02 ± 0.07^ab^

Data are expressed as mean ± standard error. Number of detected samples in each group is six. Means, which share the same superscript symbol(s) in the same column, are not significantly different. *CG*, control group; *AFE*, avocado fruit extract; *ASE*, avocado seed extract. *ALT*, alanine transaminase; *AST*, aspartate transaminase; *ALP*, alkaline phosphatase; *GGT*, gamma glutamyltransferase

### Effect on serum AFP, CEA, CA19.9, and miR-122 levels

Treatment with DEN/2AAF induced a marked (*P* < 0.05) elevation in serum CEA, AFP, and CA19.9 levels compared to the normal group. Rats administered DEN/2AAF treated with hydroethanolic avocado extracts (fruit and seed) observed a noticeable amelioration in serum CEA, AFP, and CA19.9 levels. The avocado seed extract showed a more potent improvement in the elevated serum levels of AFP and CEA. In contrast, the avocado fruit extract showed a potential effect in decreasing serum CA19.9 levels. The administration of DEN/2AAF exhibited a significant increase in serum miR-122 expression levels compared to the normal group. However, rats administered DEN/2AAF and treated with avocado fruit and seed hydroethanolic extracts revealed a marked decrease in serum miR-122 expression levels compared to the DEN/2AAF-administered group (Table 4).

**Table 4:** Effect of avocado hydroethanolic extracts on serum AFP, CEA, CA19.9, and miR-122 levels in rats administered DEN/2AAF

Groups	AFP (ng/ml)	CEA (ng/ml)	CA19.9 (U/L)	miR-122 (Relative to control)
Normal group	0.42 ± 0.030 ^a^	0.41 ± 0.008 ^a^	1.24 ± 0.06 ^a^	1.05 ± 0. 030
DEN/2AAF- CG	4.40 ± 0.101 ^d^	3.73 ± 0.363 ^c^	3.29 ± 0.34 ^b^	5.60 ± 0.513
DEN/2AAF + AFE	1.97 ± 0.0176 ^c^	1.15 ± 0.070 ^b^	1.24 ± 0.05 ^a^	2.05 ± 0.092
DEN/2AAF + ASE	1.26 ± 0.119 ^b^	0.94 ± 0.018 ^ab^	1.41 ± 0.09 ^a^	2.03 ± 0.154

Data are expressed as mean ± standard error. Number of detected samples in each group is six. Means, which share the same superscript symbol(s) in the same column, are not significantly different. *CG*, control group; *AFE*, avocado fruit extract; *ASE*, avocado seed extract. *AFP*, alpha fetoprotein; *CEA*, carcinoembryonic antigen; *CA19.9*, carbohydrate antigen 19.9

### Effect on liver oxidative stress and antioxidant defense system

The injection of DEN/2AAF into Wistar rats induced an apparent (*P* < 0.05) increase in LPO levels and a marked decrease in GSH levels compared to normal rats. However, rats administered DEN/2AAF and treated with hydroethanolic avocado extracts (fruit and seed) revealed a significant decrease in LPO levels with a noticeable increase in the GSH levels compared to the DEN/2AAF-administered group. The avocado fruit extract revealed a more potent effect than avocado seed extract in decreasing the elevated liver LPO levels and increasing the lowered GSH levels (Table 5).

**Table 5:** The effect of avocado hydroethanolic extracts on liver LPO and GSH levels and GPx, GST, and SOD activities in rats administered DEN/2AAF

Groups	LPO (nmole MDA /100 mg tissue/hr)	GSH (nmole /100 mg tissue)	GPx (mU/100 mg tissue)	GST (U/100 mg tissue)	SOD (U/g tissue)
Normal group	45.02 ± 2.23 ^a^	92.41 ± 3.31 ^c^	110.66 ± 6.36 ^b^	125.83 ± 7.98^b^	12.01 ± 0.82^c^
DEN/2AAF- CG	93.32 ± 5.28 ^b^	60.50 ± 5.44 ^a^	47.00 ± 8.09 ^a^	73.16 ± 6.22^a^	6.31 ± 0.47^a^
DEN/2AAF + AFE	47.70 ± 2.70 ^a^	85.66 ± 3.61^bc^	104.00 ± 5.34^b^	120.00 ± 5.41^b^	9.26 ± 0.45^b^
DEN/2AAF + ASE	60.06 ± 2.45 ^a^	79.33 ± 2.45 ^b^	93.50 ± 3.56^b^	114.90 ± 3.78^b^	10.07 ± 0.31^b^

Results are expressed as mean ± standard error. Each group has six samples. Means, which share the same superscript symbol(s) in the same column, are not significantly different. *CG*, control group; *AFE*, avocado fruit extract; *ASE*, avocado seed extract. *SOD*, superoxide dismutase, *GST*, glutathione-S-transferase; *GPx*, glutathione peroxidase; *GSH*, glutathione; *LPO*, lipid peroxidation

Notably, the treatment of Wistar rats with DEN/2AAF revealed a marked decrease in GPx, GST, and SOD activities compared with the normal rats. The administration of avocado fruit and seed hydroethanolic extracts revealed a significant increase in the lowered activities of SOD, GST, and GPx, as compared to DEN/2AAF-administered group. The avocado fruit extract was more effective than seed extract in improving the antioxidant enzyme activities in DEN/2AAF-administered rats (Table 5).

### Effects on liver tissue mRNA gene expressions

**Fig. 2:**
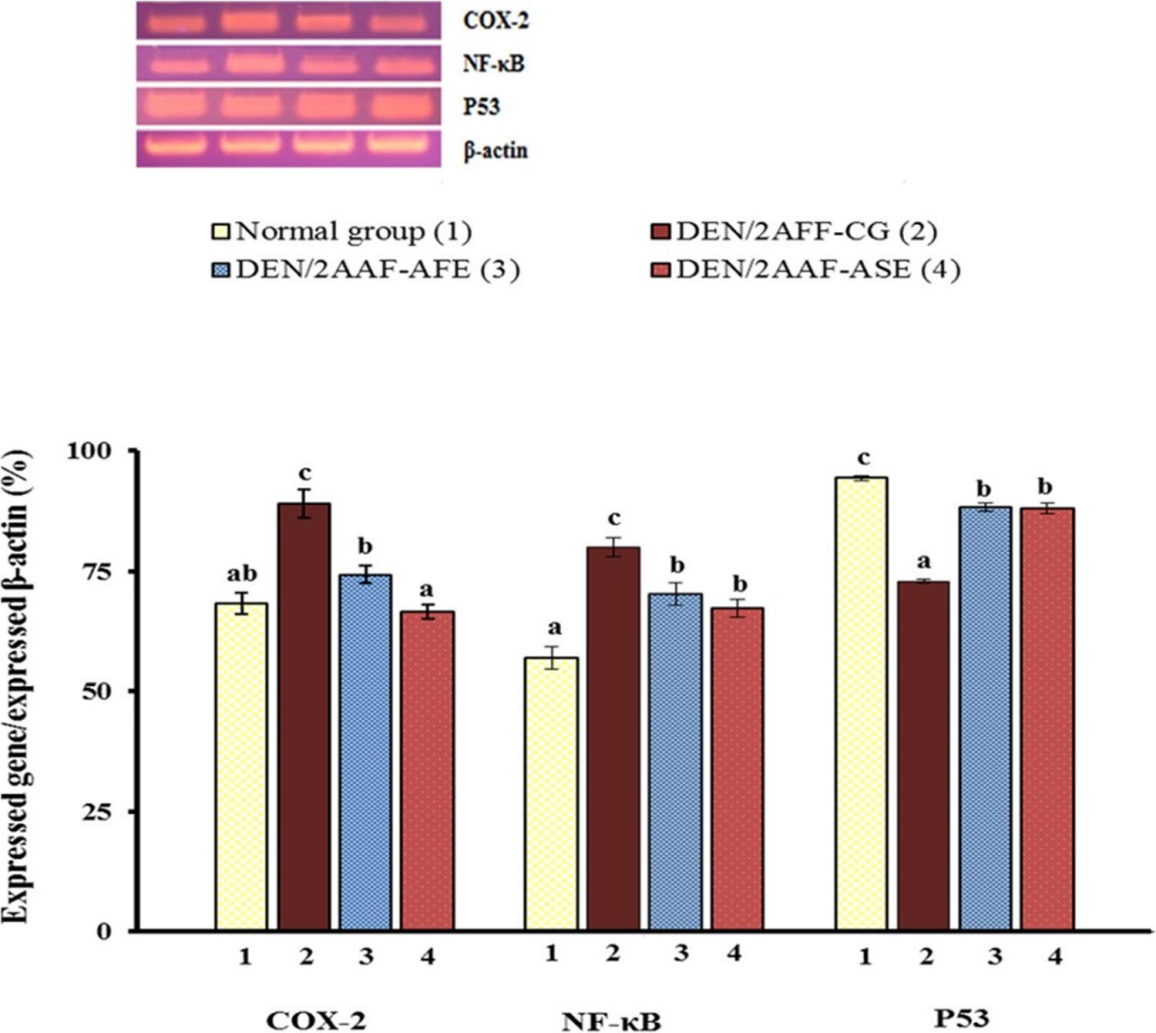
Impact of avocado fruit and seed hydroethanolic extracts on liver tissue COX-2, NF-κB, and P53 mRNA expression in DEN/2AAF-administered rats. Means which have symbols(s) are not significantly different

Administration of DEN/2AAF to Wistar rats induced a significant (*P* < 0.05) upregulation in COX-2 and NF-κB mRNA expressions relative to the normal group. DEN/2AAF-administered rats treated with avocado hydroethanolic avocado extracts observed a significant (*P* < 0.05) downregulation in the increased mRNA expressions of COX-2 and NF-κB relative to DEN/2AAF-administered group. Importantly, DEN/2AAF-administered rats showed a marked downregulation in p53 mRNA expressions relative to the normal group. The DEN/2AAF-administered group treated with avocado hydroethanolic extract exhibited a significant upregulation in p53 mRNA expressions when relative to the DEN/2AAF-administered group (Fig. 2).

### Immunohistochemical of BAX and Ki-67

**Fig. 3:**
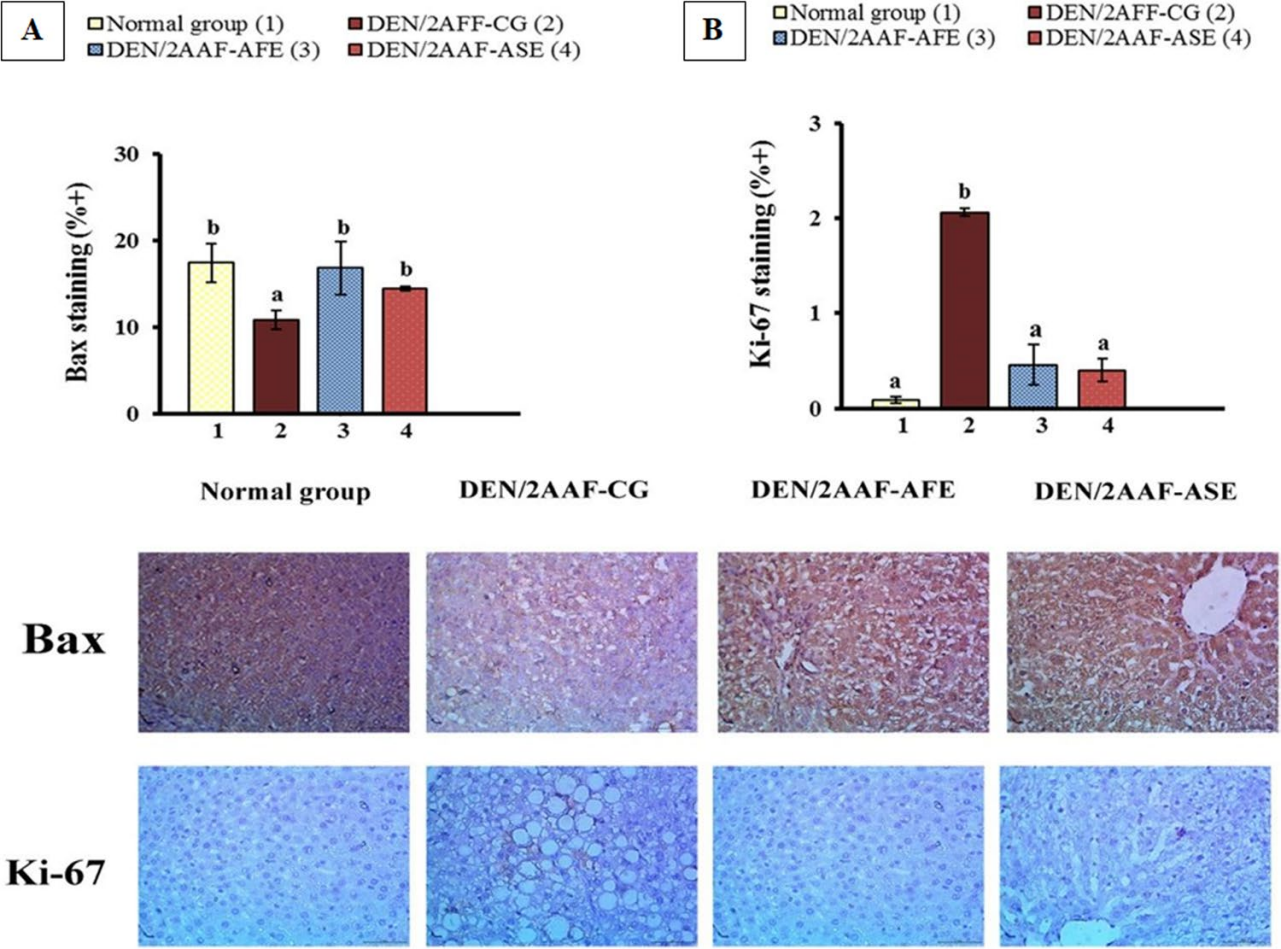
Impact of avocado fruit and seed hydroethanolic extracts on the immunoreactivity of BAX (**A**) (400 ×) and Ki-67 (**B**) (400 ×) in rats administered DEN/2AAF

There was a significant decrease in hepatic BAX protein intensity in immunohistochemistry staining than in rats exposed to DEN/2AAF relative to the normal group. Conversely, in the group treated with avocado fruit and seed hydroethanolic extracts, there was a noticeable increase in BAX intensity staining than that of the DEN/2AAF-administered group (Fig. 3). Regarding intensity staining, a significant increase in hepatic Ki-67 intensity staining was more than that of animals exposed to DEN/2AAF compared to the normal rats. Otherwise, there was a marked decrease in Ki-67 intensity staining in the group treated with avocado fruit and seed hydroethanolic extracts than in the DEN/2AAF-administered rats (Fig. 3).

### The liver histopathological changes

**Fig. 4:**
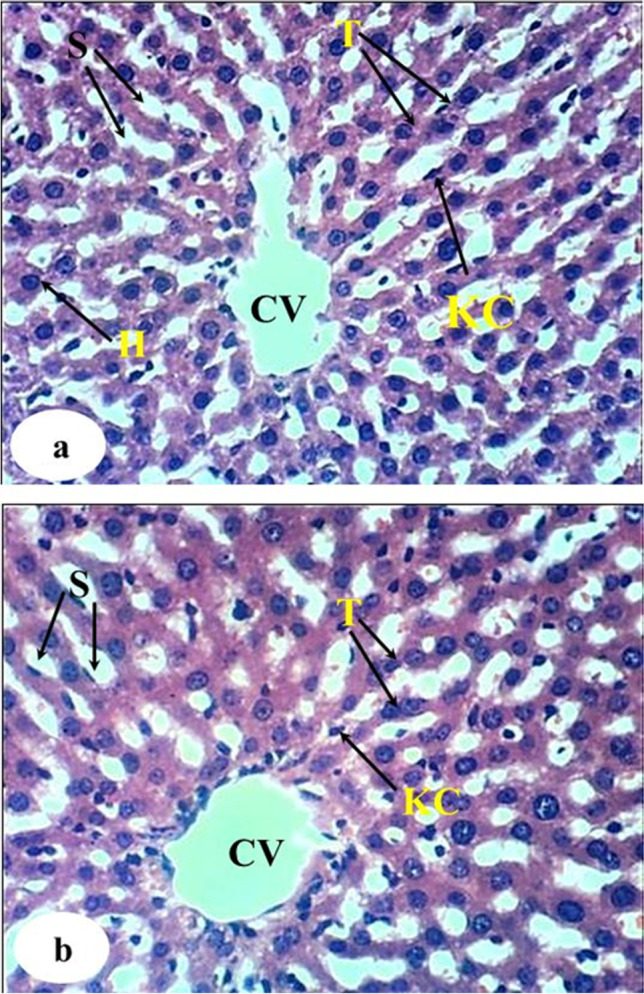
Photomicrograph of liver sections of normal rats showing the normal histological structure of hepatic lobule, the central vein, the sinusoid (S), hepatic strand or trabeculae (T), Kupffer cells, and concentrically arranged hepatocytes (H) (H & E, X400)

**Fig. 5:**
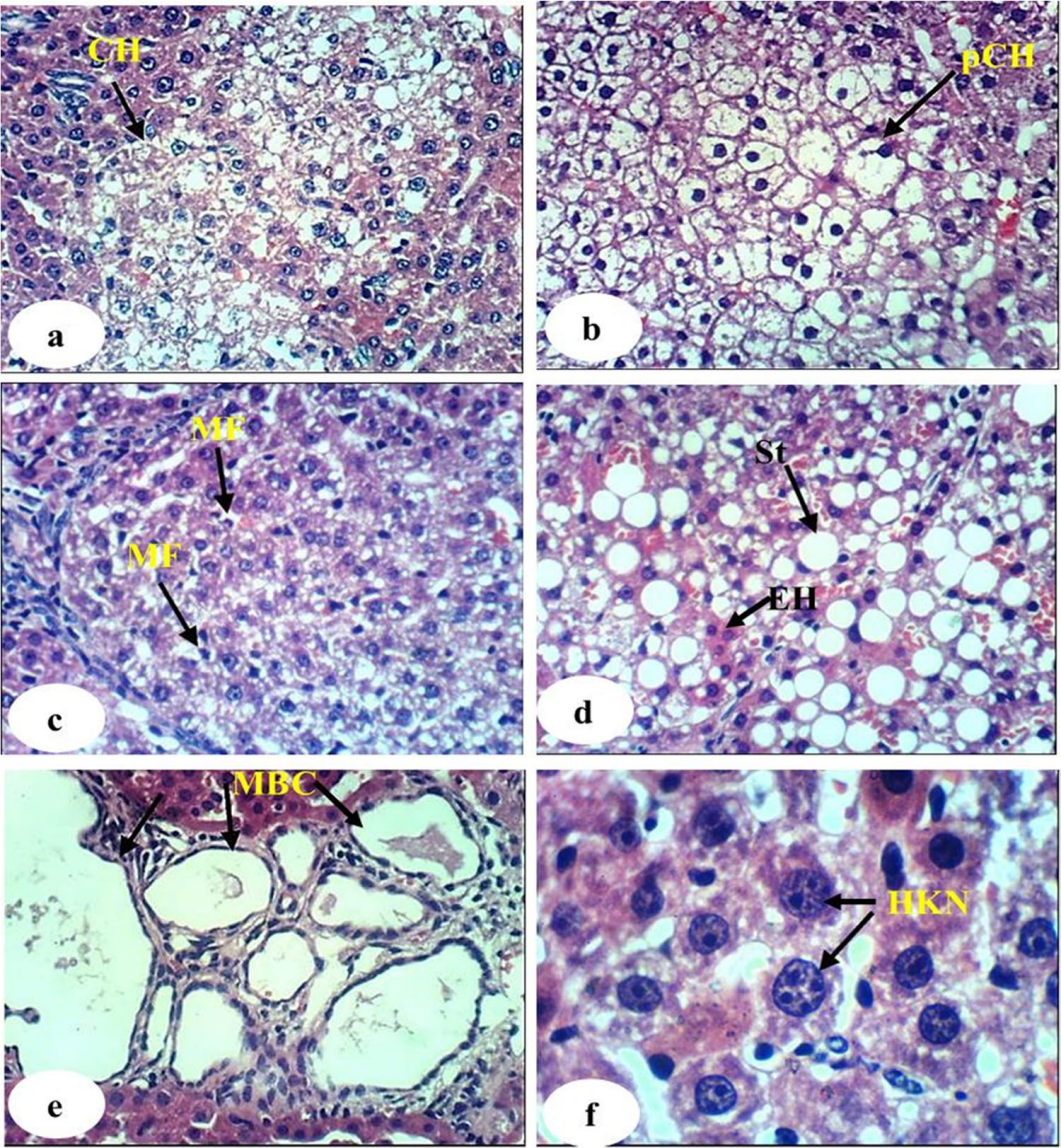
Photomicrographs of liver sections of rats administered DEN/2AAF showing proliferated clear foci of hepatocytes with active vesicular nuclei (5a, 400 ×), polyhedral clear hepatocytes (pCH) (5b, 400 ×), dysplastic, highly proliferated, vacuolated hepatocytes with mitotic figures (MF) (5c, 400 ×), steatohepatitis nodule (St) in association with highly proliferated eosinophilic hepatocytes (EH) (5d, 400 ×), multiple biliary cysts in association with highly proliferated hepatocytes (5e, 400 ×), and hyperchromatic karyomegalic nuclei with prominent more than one nucleolus (5d, 1000 ×)

**Fig. 6:**
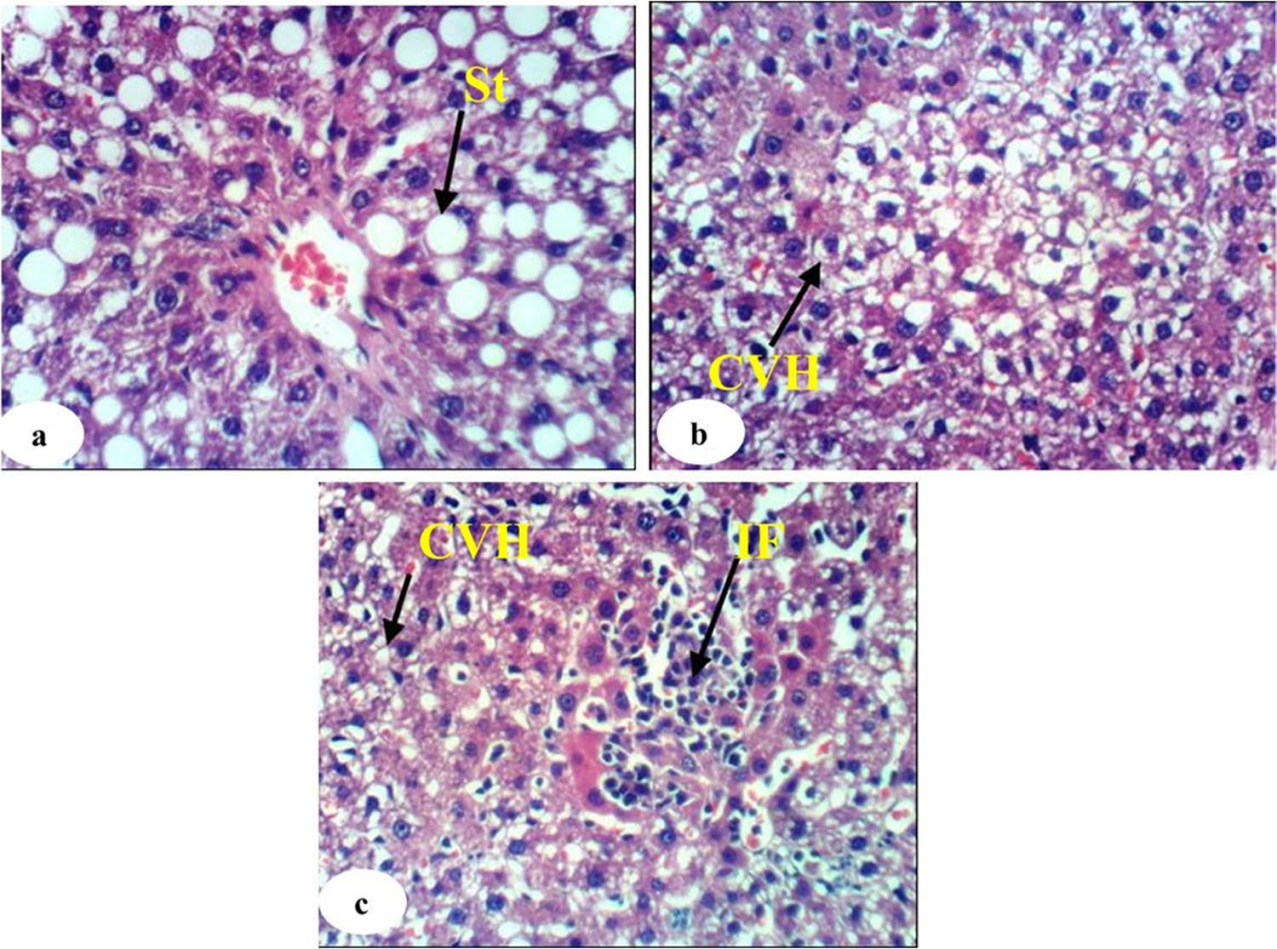
Photomicrographs of the liver sections of rats administered DEN/2AAF and treated with avocado fruit extract showing hepatic steatosis (St) (6a, 400 ×), cytoplasmic vacuolization of hepatocytes (CVH) (6b, 400 ×), and focal hepatic necrosis associated with inflammatory cells infiltration (IF) (6c, 400 ×)

**Fig. 7:**
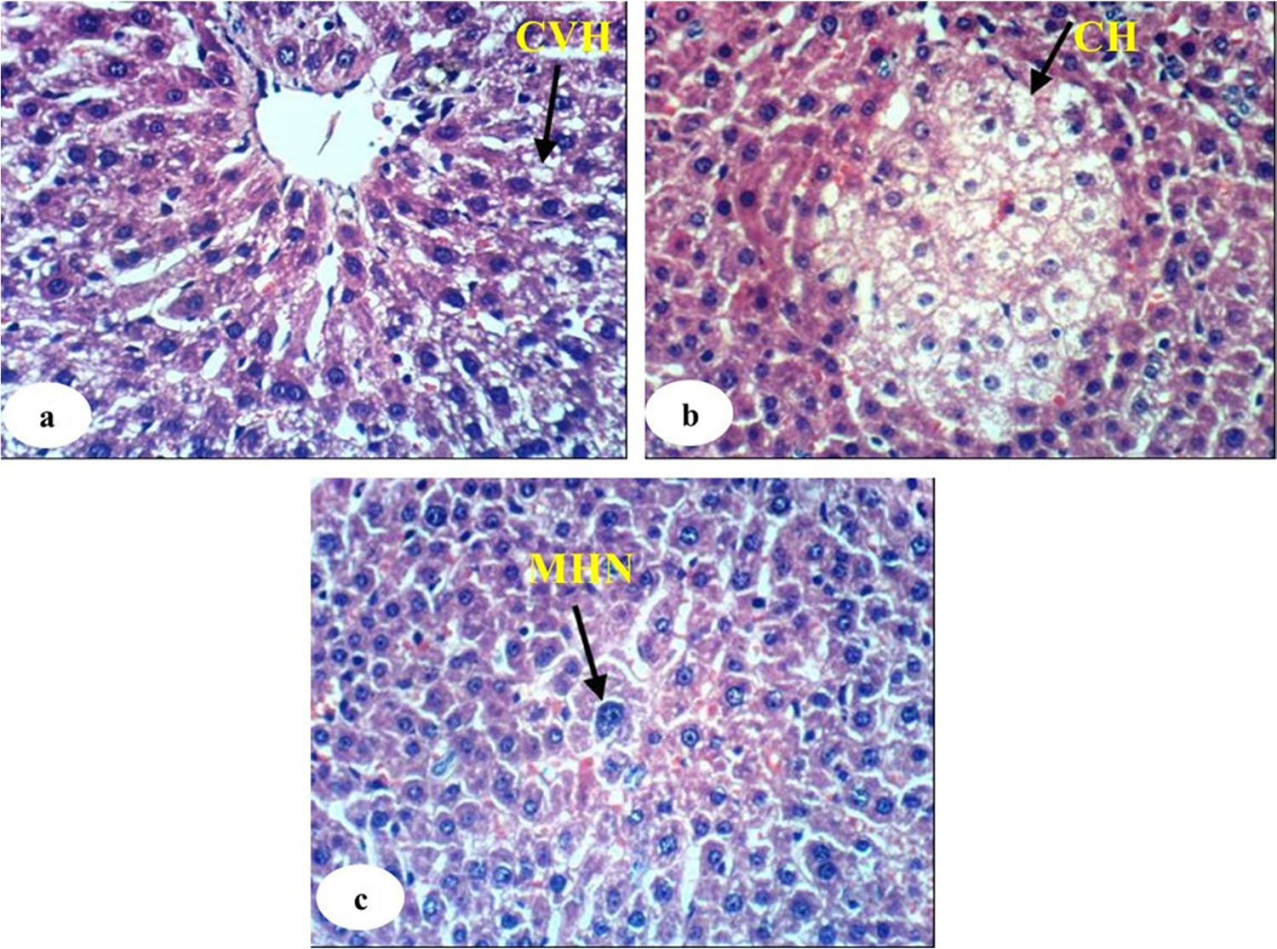
Photomicrographs of the liver sections of DEN/2AAF rats treated with avocado seed extract showing cytoplasmic vacuolization of hepatocytes (CVH) (7a, 400 ×) and focal hepatic necrosis associated with inflammatory cell infiltration (IF) (7b, 400 ×), and some dysplastic proliferated hepatocytes with karyomegallic, misshaped, and hyperchromatic nuclei (MHN) (7c, 400 ×)

The histological examination of normal liver, DEN/2AAF-administered control, rats administered DEN/2AAF and treated with avocado fruit extract, and rats administered DEN/2AAF and treated with avocado seed extract was observed in Figs. 4, 5, 6, and 7, respectively. The liver sections of the normal group showed a normal organized histological structure of the hepatic lobule and normal hepatocytes forming the hepatic cords (trabeculae), radiating from the central vein toward the periphery and alternate with narrow blood spaces known as sinusoids that are lined with Kupffer cells (Fig. 4a and b). The liver of rats administered DEN/2AAF showed cancerous lesions characterized by proliferated clear foci of hepatocytes with active vesicular nuclei (Fig. 5a); polyhedral clear hepatocytes (Fig. 5b); dysplastic, heavily proliferated, and vacuolated hepatocytes mitotic figures (Fig. 5c); parenchymatous steatohepatitis nodule in association with heavily proliferated eosinophilic hepatocytes (Fig. 5d); multiple biliary cysts in association with heavily proliferated hepatocytes (Fig. 5e); and large hyperchromatic karyomegalic nuclei with more than one prominent nucleolus (Fig. 5d). The liver of rats administered DEN/2AAF and treated with avocado fruit extract showed marked improvement as compared to DEN/2AAF-administered control but still depicted hepatic steatosis (Fig. 6a), cytoplasmic vacuolization of hepatocytes (Fig. 6b), and focal hepatic necrosis associated with inflammatory cell infiltration (Fig. 6c). Similarly, the liver of rats administered DEN/2AAF and treated with avocado seed extract exhibited marked amelioration but still have cytoplasmic vacuolization of hepatocytes (Fig. 7a) and focal hepatic necrosis associated with inflammatory cell infiltration (Fig. 7b) as well as some dysplastic proliferated hepatocytes with karyomegallic, misshaped, and hyperchromatic nuclei (Fig. 7c).

## Discussion

The previous data indicated that DEN played a crucial role in inducing liver carcinogenesis through increased ROS generation and decreased liver antioxidant enzymes (Sivaramakrishnan et al. 2008). Elevated levels of prooxidants and different oxidative stress biomarkers induced significant damage in cells and tissues associated with cancer pathogenesis (Kruk et al. 2019). In our study, DEN/2AAF administration to rats led to a marked increase in enzymes activities of serum liver function (GGT, ALP, ALT, and AST) compared to the normal group. The current results agree with Mohamed and Amr (2013) and Gouegni and Abubakar (2013), who reported that liver function enzyme activities were significantly reduced after administration of avocado extracts. In our study, an elevated level of serum bilirubin and decreased total serum protein and albumin levels were observed in DEN/2AAF-administered rats compared to normal rats. The treatment of hydroethanolic avocado fruit and seed extracts improved total serum protein and albumin levels. Furthermore, oil extracted from avocado fruit revealed the beginning of the regeneration of liver function, thereby helped to increase total protein and albumin levels (Carvajal-Zarrabal et al. 2014). In this regard, avocado fruit extract improved the liver function of DEN-administered rats due to the protective antioxidant mechanisms employed by both enzymatic and nonenzymatic substances such as phenolic and flavones (Rainey et al. 1994).

AFP is a well-known basic marker that increases in liver cancer, and CEA is also important in case of metastases to this organ (Snarska et al. 2006). Thus, AFP and CEA are effective biomarkers elevated in primary HCC and metastatic HCC (Qi et al. 2020). Importantly, AFP, CA19-9, and CEA together could help diagnose asymptomatic patients with primary hepatic cancer (Alhadi et al. 2019). In our study, the DEN/2AAF-administrated control group showed elevated liver tumor markers (CEA, AFP, and CA19.9) compared to the normal group. Numerous studies have shown that rat exposure to certain carcinogens, such as DEN, causes elevated levels of AFP (Sivaramakrishnan et al. 2008; Singh et al. 2009). Increased CEA and AFP levels support the incidence of HCC in the control group of DEN/2AAF; this finding is consistent with Gokuladhas et al. (2015), who reported that DEN induced an increase in HCC biomarkers.

MicroRNAs (miRNAs) are unique biomolecules that are exceptionally persistent against destruction (Link and Goel 2013). The miR-122 has been reported to have higher levels in serum of patients with HCC compared with healthy controls by various publications (Xu et al. 2011; Karakatsanis et al. 2013; El-Garem et al. 2014; Li et al. 2019). This study found that serum miR-122 was significantly elevated in DEN/2AAF-administered groups compared to the normal group. MiR-122 may be released into the bloodstream during hepatocellular injury due to its high expression in liver tissue (Hou et al. 2011). A possible relationship between AFP and miR-122 has been hypothesized in a mouse model (Ambade et al. 2016). The supplementation of hydroethanolic avocado fruit and seed extracts to rats administered DEN/2AAF significantly reduced the elevated serum AFP, CEA, CA19.9, and miR-122 levels indicating the anticancer activity of avocado treatments. These findings agree with Larijani et al. (2014), who described that avocado fruit extract is rich in phytochemicals that had a significant role in inhibiting cancer cell growth. Also, avocado seed extract is richer in phenolic compounds that are potential anticancer agents (Platas et al. 2012). These elucidations are supported by Kushi et al. (2006), who stated that some avocado seed phytochemicals have anticarcinogen properties by blocking the action of carcinogens on their target organs and suppress cancer development.

Importantly, DEN/2AAF administration in rats induced an increase in LPO levels (Nafees et al. 2013), supported by the findings of Ahmed et al. (2019). LPO elevations induced a loss of the membrane property, and their reactive products may also damage other molecules (Thanan et al. 2015). In our research, enzymatic antioxidant (GPx, GST, and SOD) and nonenzymatic antioxidant (GSH) activities were decreased in rats administered DEN/2AAF compared to normal rats. Our data is consistent with Gokuladhas et al. (2015) results, who reported a reduction in the tissue antioxidants (enzymatic and nonenzymatic) in Wistar rats administered DEN. On the other side, the current investigation showed that hydroethanolic extracts of avocado fruit and seed supplementation reduced the liver LPO in rats administered DEN/2AAF. This result agreed with Hamouda et al. (2016), who suggested that avocado extract can protect the liver against oxidative stress, possibly through the antioxidant effects of its bioactive compounds.

In addition, there was a significant increase in enzymatic and nonenzymatic antioxidants in the liver of rats administered DEN and treated with hydroethanolic extracts of avocado fruits and seeds. These bioactive substances in avocado fruit extract have an antioxidant and free radical suppressing effect (Duester 2001). Notably, Mohamed and Amr (2013) reported an increase in liver GSH content and activities of GPx and SOD in carbon tetrachloride–intoxicated rats supplemented with avocado fruits. Similarly, phenolic compounds found in avocado seed extract were strong antioxidant agents because they could protect the human body from free radicals (Mushtaq and Wani 2013). Our results are consistent with the data reported by Sadek et al. (2012), who indicated that rats treated with avocado fruit and seed extracts observed a substantial increase in glutathione material compared to control rats. Notably, GC–MS is a valuable tool for the reliable identification of phytocompounds (SampathKumar & RamaKrishnan 2011). GC–MS analysis of avocado fruit and seed hydroethanolic extracts showed phytocomponents, like 5-Hydroxymethylfurfural (5-HMF), 9-Octadecyne, and 9-octadecenamide. Previous results showed that 5-HMF, 9-Octadecyne, and 9-octadecenamide exhibited novel antioxidant activities (Cheng et al. 2008; Chew et al. 2009; Zhao et al. 2013). Also, 5-HMF displayed higher antiproliferative activities on human melanoma (Zhao et al. 2013).

Our data showed a significant increase in COX-2 mRNA expression in the liver of rats administered DEN/2AAF compared to normal rats. These data agree with Zhang et al. (2017), who found that DEN induces inflammation via oxidative-dependent way involving ROS and activated COX-2. Consistently, Chen et al. (2017) reported that COX-2 expression is upregulated markedly in HCC. In the current study, the data showed a marked increase in the levels of NF-κB mRNA expression in the liver of rats administered DEN/2AAF relative to normal rats. Consistent with our results, Sahin et al. (2014) found that DEN-induced inflammation by ROS subsequently activated NF-κB, followed by the release of carcinogenic substances that led to HCC development.

Interestingly, the treatment of rats administered DEN/2AAF with hydroethanolic extracts from avocado fruits and seeds lowered the elevated COX-2 and NF-κB mRNA expression, reflecting the antiinflammatory and anticancer efficacy of these treatments against hepatocarcinogenesis. Avocado fruit extract contains phytochemicals with potent free radical scavengers, including carotenoids (lutein, zeaxanthin, and α- and β-carotene) (Dreher and Davenport 2013). Additionally, β-Carotene, commonly found in natural foods, reduces the inflammation mediated by COX-2 and NF-κB in various disease signals (Aravindaram and Yang 2010). Furthermore, avocado seed extract is rich in polyphenols with antioxidant and antimicrobial activities (Rodríguez-Carpena et al. 2011), which have the same effect on COX-2 and NF-κB. Our findings agreed with Hamouda (2015) data, who reported that rats administered DEN and treated with avocado fruit extract showed a significant decrease in COX-2 protein levels.

Apoptosis is a cancer chemoprevention process that eliminates the cells undergoing a neoplastic transformation in conditions where other defense mechanisms fail to obstruct carcinogenesis (Taha et al. 2009). The proapoptotic BAX protein is mainly involved in regulating the intrinsic pathway of apoptosis. The p53 gene is a tumor suppressor gene that activates BAX to cause apoptosis (Kelly and Strasser 2011). In response to DNA damage and oncogene activation, p53 plays an essential role in cellular stress response programs (Vousden and Prives 2009). According to several studies, p53 mutations are found in 30–60% of HCC cases (Hussain et al. 2007). The present investigation showed a significant decrease in p53 and BAX levels due to DEN/2AAF administration in rats. These results agreed with He et al. (2016), who reported that p53 mutations are among the most common genetic alterations in human cancers, including HCC. Markedly, DEN decreases BAX expression in the liver of carcinogenic rats (Ou et al. 2015).

On the other hand, avocado fruit and seed hydroethanolic extracts elevated mRNA expression of p53 and BAX levels compared to the DEN/2AAF control group. In addition, Bonilla-Porras et al. (2014) reported that avocado extracts act as a proapoptotic compound and the current result indicated that avocado extracts can activate p53 and BAX expression. Besides, apoptosis appears to be an important target for avocado phytochemicals to selectively eliminate cancer cells from normal tissues (Ding et al. 2007). BAX and p53 are important mediators in the intrinsic pathway of apoptosis; their upregulation as a result of treatments of DEN/2AAF-administered rats with avocado fruit and seed extracts may indicate the possible role of apoptosis in the anticancer effects of these treatment agents.

Cell proliferation is recognized to play a pivotal role in the various stages of cancer development. Because Ki-67 is a protein with an expression pattern fully dependent on cell proliferation, its expression is usually utilized as a cell proliferation indicator (Shi et al. 2015). In the current investigation, DEN/2AAF-induced hepatocarcinogenesis was associated with an increase in Ki-67 levels. These results followed previous research that revealed Ki-67 was overexpressed in DEN-induced hepatocarcinogenesis (Raghunandhakumar et al. 2013). Treatment with avocado fruit and seed shows depletion in Ki-67 levels. The biological effects of flavonoids include scavenging free radicals, NO production regulation, apoptosis induction, and cell proliferation inhibition (Yang et al. 2001).

## Conclusions

**Fig. 8:**
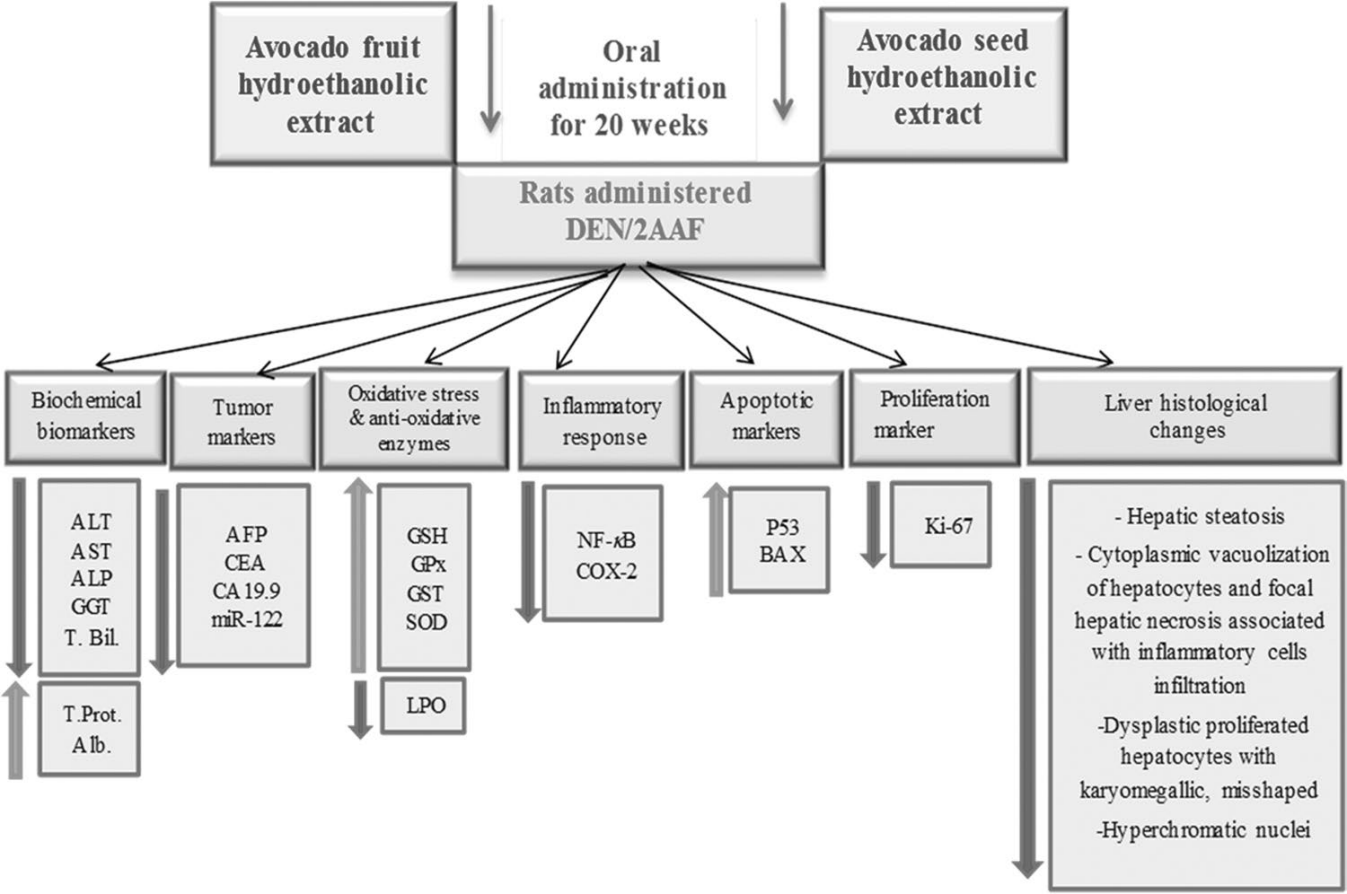
Representative summary for the probable effects of avocado hydroethanolic extracts on hepatocarcinogenesis

In conclusion, administration of hydroethanolic extracts from avocado fruit and seed to rats administered DEN/2AAF showed a decrease in liver COX-2, NF-κB, and Ki-67 levels and an increase in liver p53 and BAX levels, indicating attenuation in the liver inflammation with enhanced apoptosis pathway. Thus, hydroethanolic avocado extracts may have chemopreventive effects against DEN/2AAF-induced hepatocarcinogenesis and hepatocarcinoma via stimulation of antioxidant defense system, inhibiting inflammatory response, suppressing cell proliferation, and stimulating apoptosis. Proposed impacts of avocado hydroethanolic extracts on hepatocarcinogenesis are summarized in Fig. 8.

## Data Availability

This published article includes all of the data generated or analyzed during this investigation.
